# Development and Validation of a Novel Radiomics-Based Nomogram With Machine Learning to Preoperatively Predict Histologic Grade in Pancreatic Neuroendocrine Tumors

**DOI:** 10.3389/fonc.2022.843376

**Published:** 2022-03-31

**Authors:** Xing Wang, Jia-Jun Qiu, Chun-Lu Tan, Yong-Hua Chen, Qing-Quan Tan, Shu-Jie Ren, Fan Yang, Wen-Qing Yao, Dan Cao, Neng-Wen Ke, Xu-Bao Liu

**Affiliations:** ^1^ Department of Pancreatic Surgery, West China Hospital, Sichuan University, Chengdu, China; ^2^ Department of West China Biomedical Big Data Center, West China Hospital, Sichuan University, Chengdu, China; ^3^ Department of Radiology, West China Hospital, Sichuan University, Chengdu, China; ^4^ Department of Pathology, West China Hospital, Sichuan University, Chengdu, China; ^5^ Department of Oncology, West China Hospital, Sichuan University, Chengdu, China

**Keywords:** pancreas, pancreatic neuroendocrine tumor, tumor grade, radiomics, CT

## Abstract

**Backgroud:**

Tumor grade is the determinant of the biological aggressiveness of pancreatic neuroendocrine tumors (PNETs) and the best current tool to help establish individualized therapeutic strategies. A noninvasive way to accurately predict the histology grade of PNETs preoperatively is urgently needed and extremely limited.

**Methods:**

The models training and the construction of the radiomic signature were carried out separately in three-phase (plain, arterial, and venous) CT. Mann–Whitney *U* test and least absolute shrinkage and selection operator (LASSO) were applied for feature preselection and radiomic signature construction. SVM-linear models were trained by incorporating the radiomic signature with clinical characteristics. An optimal model was then chosen to build a nomogram.

**Results:**

A total of 139 PNETs (including 83 in the training set and 56 in the independent validation set) were included in the present study. We build a model based on an eight-feature radiomic signature (group 1) to stratify PNET patients into grades 1 and 2/3 groups with an AUC of 0.911 (95% confidence intervals (CI), 0.908–0.914) and 0.837 (95% CI, 0.827–0.847) in the training and validation cohorts, respectively. The nomogram combining the radiomic signature of plain-phase CT with T stage and dilated main pancreatic duct (MPD)/bile duct (BD) (group 2) showed the best performance (training set: AUC = 0.919, 95% CI = 0.916–0.922; validation set: AUC = 0.875, 95% CI = 0.867–0.883).

**Conclusions:**

Our developed nomogram that integrates radiomic signature with clinical characteristics could be useful in predicting grades 1 and 2/3 PNETs preoperatively with powerful capability.

## Introduction

Pancreatic neuroendocrine tumor (PNET) is a relatively rare pancreatic disorder thought to arise in hormone secretory cells of the islets of Langerhans (1) and ever known as islet cell tumor (2). It consists of about 3%–5% of all the pancreatic neoplasm but predominates human neuroendocrine tumors (3). Additionally, the incidence and prevalence of PNETs are steadily increasing in recent decades (4, 5).

PNETs are characterized by tumor heterogeneity (6), and of which the clinical behavior are relatively indolent but vary dramatically (7). Tumor grade is the crucial determinant of the biological aggressiveness of PNETs. Additionally, it is suggested to be associated with lymph node involvement (7), tumor recurrence (8), and overall prognosis (9). According to the 2010 World Health Organization (WHO) classification criteria (10), tumor grade is defined numerically by the proliferative indicator Ki-67, in which low-grade (grade 1 (G1)) tumors have a Ki-67 index from 0% to 2%, intermediate-grade (G2) tumors have a Ki-67 index from 3% to 20%, and high-grade (G3) tumors have a Ki-67 index greater than 20%. Surgery is thought to be the cornerstone of treatment of PNETs in each stage and the only potential way to cure local PNETs (5, 11). However, different surgical strategies could be applied for PNETs of grades 1 and 2/3. The last but not the least, for advanced PNETs, there are also other treatment options, e.g., somatostatin analog (SSA), targeted therapy, or chemotherapy, based on tumor grades. In short, the WHO tumor grading is the best current tool to predict prognosis, guide therapy selection, and aid surgical decision-making by stratification of PNETs.

Of note, tumor grade is always obtained according to postoperative pathology specimen. Although the preoperative endoscopic ultrasound (EUS)-guided fine-needle aspiration cytology (FNA) is proved to be efficient in diagnosing PNETs, the accuracy in differentiating tumor grade remains challenging, possibly due to limited tissue availability or missing the most mitotically active areas (hot pot) of the tumor. The research of Heidsma et al. showed that tumor grade differentiation could be accurately determined by FNA in only 20%–50% of patients (6, 12). Additionally, EUS-guided fine-needle biopsy (FNB) with thicker tissue biopsy needle was reported to have better performance in tumor grade differentiation, as more tumor tissues could be obtained (13). However, both of them were invasive procedures which largely depended on the operators’ experience (13). Therefore, the effective method of preoperatively predicting the pathologic grade of PNETs is still imperatively needed to help establish individualized therapeutic strategies and aid surgical decision-making.

Several previous studies tried to identify the tumor grade of PNETs by computed tomography (CT), magnetic resonance imaging (MRI), and PET/CT (14–17). Although they provided a noninvasive way to preoperatively predict the aggressiveness of PNETs, the accuracy was limited, as the prediction of the frequently occurring heterogeneous tumor was mainly established based on visual observation rather than quantitative information. Recently, “radiomics” brings a new hope for this problem. It is a method that automatically extract a large number of quantitative features from medical images using data-characterization algorithms, and subsequently identify the most significant radiomic signatures through machine learning methods (18, 19). Therefore, we can realize cancer detection, prediction of clinical outcome, and treatment evaluation as reported previously (20, 21). Additionally, radiomics was reported to be successfully applied in differentiating pathologic grading in patients with clear cell renal cell carcinoma (22), colorectal adenocarcinoma (23), etc. Nevertheless, to the best of our knowledge, a noninvasive optimal combined model to incorporate imaging features with clinical characteristics (such as tumor size and tumor margin status) to predict the pathologic grade of PNETs is extremely limited.

Thus, this work attempted to establish a multimodal artificial intelligence (AI) model that integrates a radiomic signature based on plain CT images with clinical features for noninvasive and preoperative prediction of the pathologic grades of PNETs.

## Materials and Methods

### Patients

This retrospective study was approved by the ethics committee of Sichuan University, and the signed informed consent was waived. From July 2008 to June 2018, patients with histologically confirmed PNETs who underwent surgical resection in our institution were retrospectively reviewed. The patients with a PNET that was too small to display clearly on CT, several patients with cystic PNET, and patients without preoperative CT scan were excluded at the present study. The final diagnosis of PNETs was made by specialized pathologists, including the diagnosis of the tumor grade basing on Ki-67 immunohistochemical staining data. Clinical data were obtained from the electronic medical records or external medical reports, including demographic characteristics and classification. Finally, 139 patients with complete data available were identified for analysis in the present study. Of these, 83 patients were taken randomly as the training set, and the other 56 patients were used for the independent validation set (also called test set, not the set in a crossvalidation approach). The training dataset and validation dataset had an even distribution in patient characteristics (**Table 1**). No significant difference was found in PNET pathologic grade and clinical characteristics (age, maximum diameter, and clinical stage of the tumor, etc.) between the training dataset and validation dataset.

**Table 1:** Comparison of patient and lesion features between grades 1 and 2/3 groups in training and validation sets.

Features	Training set (*n* = 83)		*p*-value	Validation set (*n* = 56)		*p*-value
	Grade 2/3 (*n* = 55, %)	Grade 1 (*n* = 28, %)		Grade 2/3 (*n* = 37, %)	Grade 1 (*n* = 19, %)	
Age(range, years)^a^	49.7 (20–77)	49.2 (24–70)	0.883	52 (22–77)	53.2 (16–75)	0.799
**Gender**			0.141			
**Women**	27 (49.1)	9 (32.1)		10 (27)	5 (26.3)	0.955
**Men**	28 (50.9)	19 (67.9)		27 (73)	14 (73.7)	
Tumor size (range, mm)^a^	40.7 (12–150)	28.4 (10–80)	**0.028**	47.7 (12–180)	22.2 (12–42)	**<0.001**
**T stage (T3–T4)**	28 (50.9)	5 (17.9)	**0.004**	23 (62.2)	Nil	**<0.001**
**Clinical TNM stage (IIB and above)**	31 (56.4)	5 (17.9)	**0.001**	25 (67.6)	1 (5.3)	**<0.001**
**Dilated MPD/BD^b^**	19 (34.5)	3 (10.7)	**0.02**	14 (37.8)	Nil	**0.006**
**Tumor margin**			**0.013**			**0.034**
**Well defined**	30 (54.5)	23 (82.1)		24 (64.9)	18 (94.7)	
**Poorly defined**	25 (45.5)	5 (17.9)		13 (35.1)	1 (5.3)	
**Tumor location**			0.502			0.096
**Head and neck**	29 (52.7)	14 (50)		20 (54.1)	6 (31.6)	
**Body and tail**	26 (47.3)	13 (46.4)		14 (37.8)	13 (68.4)	
**Multiple**	Nil	1 (3.6)		3 (8.1)	Nil	
**Pathology**			0.175			0.080
**Functional**	12 (21.8)	10 (35.7)		9 (24.3)	9 (47.4)	
**Nonfunctional**	43 (78.2)	18 (64.3)		28 (75.7)	10 (52.6)	
**Insulinoma**			**0.047**			**0.006**
**Yes**	5 (9.1)	8 (28.6)		4 (10.8)	9 (47.4)	
**No**	50 (90.9)	20 (71.4)		33 (89.2)	10 (52.6)	

a The values indicated are expressed as median (range). ^b^Dilated MPD/BD, dialated main pancreatic duct (MPD) or bile duct (BD). The clinical TNM stage and T stage of the tumor was determined preoperatively according to the American Joint Committee on Cancer TNM Staging System Manual, 7th edition. The bold values in this table are p-value less than 0.05, which means the features between grade 1 and 2/3 groups are significantly different.

### CT Image Acquisition

All patients underwent an abdominal contrast-enhanced CT scan preoperatively. Contrast-enhanced CT scan was performed on three CT scanners including a 16-slice CT (Toshiba Medical Systems, Japan), a 64-, and a 256-slice CT (Philips Healthcare, Netherlands). CT scans used the same CT scanning parameters: tube voltage of 120 kVp, tube current of 125 to 300 mAs, pitch of 0.6 to 1.25 mm, slice thickness of 3 to 5 mm, and reconstruction interval of 3 to 5 mm.

### Radiomic Analysis

**Figure 1:**
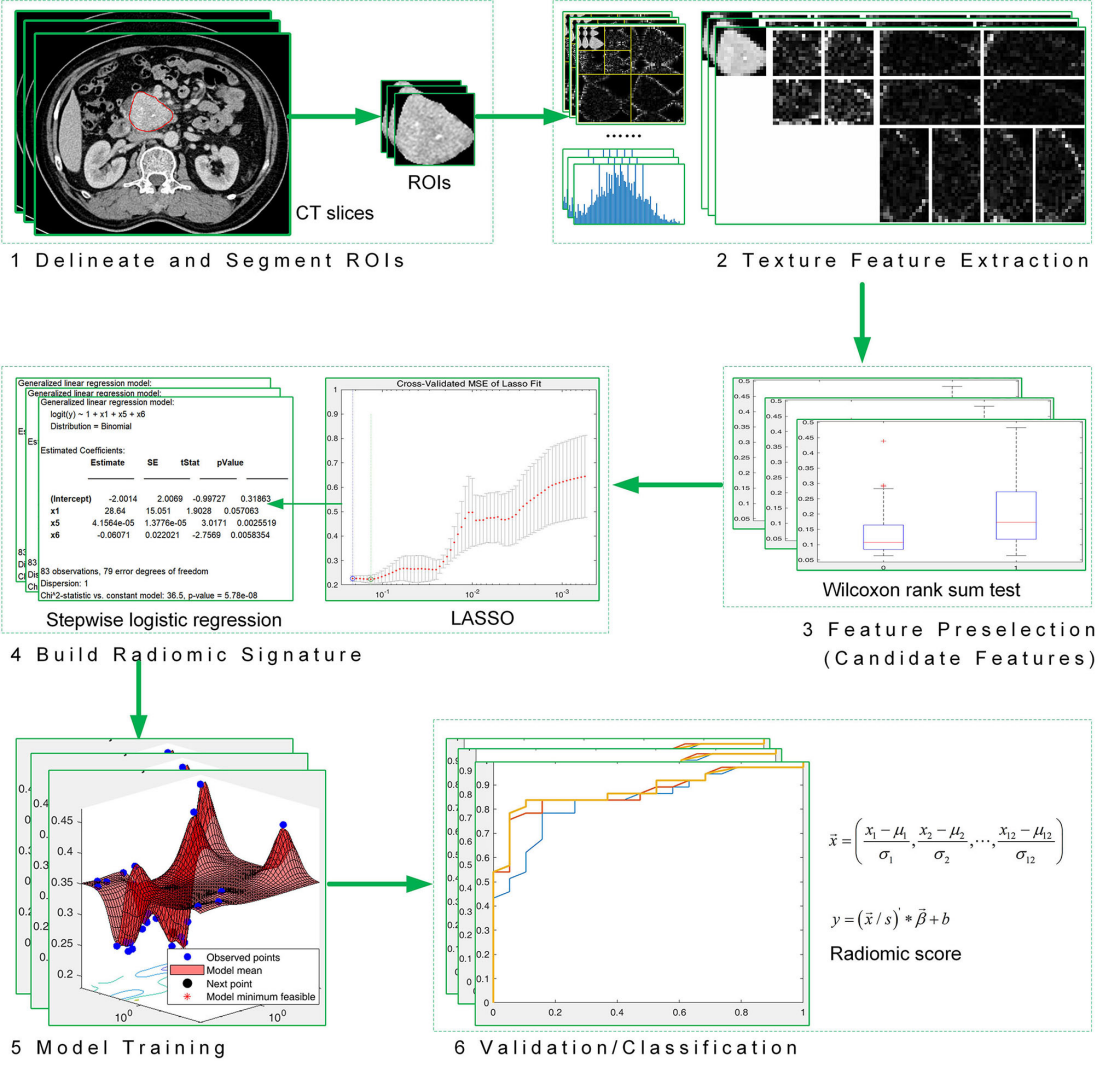
Work flow of radiomic analysis.

We performed a radiomic analysis on preoperative CT images to evauate the pathologic grades of PNETs. **Figure 1** illustrates the work flow of the radiomic analysis.

Step 1: Tumor regions were delineated and segmented into regions of interest (ROIs) from which texture features were extracted. We evaluated the CT images in plain, arterial, and portal venous phases, respectively.
Step 2: We used 10 texture analysis methods to extract features. The **Supplementary Material** described the methods in detail. A total of 1,133 features were extracted from a ROI (24–26).
Steps 3–4: Preselection was performed on the 1,133 features using the Mann–Whitney *U* test (*p*-value ≤ 0.25). We then combined the methods of least absolute shrinkage and selection operator (LASSO) and stepwise logistical regression to perform feature selection. Feature preselection and feature selection were both performed on the training set. A radiomic signature can be built based on the final selected features.
Step 5: We combined the radiomic signature and four clinical data to train SVM-linear models. Features were divided into three groups: radiomic signature (group 1), radiomic signature combining T stage and dilated main pancreatic duct (MPD)/bile duct (BD) (group 2), and radiomic signature combining T stage, dilated MPD/BD, clinical TNM stage, and tumor margin (group 3). The models training and the construction of the radiomic signature were carried out separately in three phases (plain, arterial, and venous). Thus, a total of 9 prediction tasks were performed.
Step 6: The independent validation dataset (*n* = 56) was tested on the 9 trained models. We chose an optimal model to construct a nomogram, and then used the nomogram to predict the pathologic grades of these 56 patients. A calibration curve and a goodness of fit to the ideal model are calculated to evaluate the nomogram.

## Results

The detailed distribution of clinical characteristics in the G1 group (grade 1) and G2/3 group (grade 2/3) is summarized in **Table 1**. The tumor size of PNET in grade 2/3 group was significantly larger than that in grade 1 group (*p* = 0.028). T stage (T3–T4), clinical TNM stage (IIB and above), Dilated MPD/BD, and poorly defined tumor margin were more frequently detected in patients with grade 2/3 PNETs than those with grade 1 (*p* = 0.004, *p* = 0.001, *p* = 0.02, and *p* = 0.013, respectively). The consistent results occurred both in the training and validation datasets.

As illustrated in **Figure 1**, this study aims to build a radiomic> signature and evaluate the ability of the signature to predict PNET grades. **Table 2** shows the features used to build the radiomic signature, that is, the result of feature selection on the training set. We also evaluated the performance of combining the radiomic signature and 4 clinical variables to predict PNET grades. The clinical variables are *x*_9_ to *x*_12_ in **Table 2**.

**Table 2:** Radiomic signature and clinical data.

No.	Analysis method	Subband	Feature name
*x*_1_	Fistogram		Variance
*x*_2_	Wavelet-COM	H1	Maximal correlation coefficient
*x*_3_	Wavelet-RLM	D1	Short-run low gray-level emphasis
*x*_4_	Wavelet-COM	D2	Sum of squares
*x*_5_	Contourlet-histogram	L2-1	1% percentile
*x*_6_	Contourlet-COM	L2-2	Cluster shade
*x*_7_	Contourlet-histogram	L2-3	99% percentile
*x*_8_	Contourlet-histogram	L1-2	90% percentile
*x*_9_			T stage
*x*_10_			Dilated MPD/BD
*x*_11_			Clinical TNM stage
*x*_12_			Tumor margin

The radiomic signature are composed of x_1_ to x_8_. The clinical data in group 2 are composed of x_9_ and x_10_. The clinical data in group 3 are composed of x_9_ to x_12_. The number following A, H, V, or D represents the decomposition level. The clinical TNM and T stages of the tumor were determined preoperatively according to the American Joint Committee on Cancer TNM Staging System Manual, 7th edition.

COM, cooccurrence matrix; RLM, run-length matrix; A (in the wavelet transform), approximate; H (in the wavelet transform), horizontal; V (in the wavelet transform), vertical; D (in the wavelet transform), diagonal; Li–j, jth component in the ith decomposition in the contourlet transform; Dilated MPD/BD, dilated main pancreatic duct (MPD) or bile duct (BD).

The linear combination of *x*_1_ to *x*_8_ in **Table 2** expresses the radiomic signature *y*. The linear combination is shown in Equations (1) and (2).


$$y=\left(\vec{x}/s\right)*\vec{\beta }+b$$

$$\vec{x}=\left(\frac{x_{1}-\mu_{1}}{\sigma_{1}},\frac{x_{2}-\mu_{2}}{\sigma_{2}},\cdots ,\frac{x_{m}-\mu_{m}}{\sigma_{m}}\right)$$

where *y* is the score of group 1 (or 2 or 3) for grade 1, −*y* is the corresponding score for grade 2/3, $\vec{x}$ is an observation comprising the *m* predictors, *s* is the kernel scale, *β* is the bias term, and the vector *β* contains the coefficients that define an orthogonal vector to the hyperplane,, and *μ_i_* and *σ_i_* are the corresponding weighted mean and weighted standard deviation for the *i*th predictor (used for standardization). When predicting the result, we inputted [−*y*, *y*] into the function softmax to obtain the probabilities that the observer belongs to the positive class (grade 2/3) and the negative class (grade 1).

**Figure 2:**
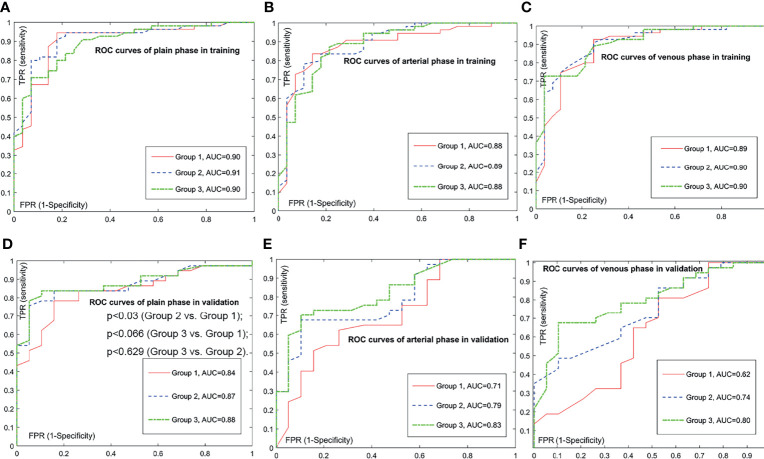
Comparison of receiver operating characteristic (ROC) curves for prediction of the histologic grade. The positive class is grade 2/3; the negative class is grade 1. Subfigure **(A–C)** illustrate the training ROCs. Subfigures **(D–F)** illustrate the validation ROCs. In validations, we performed DeLong’s tests to compare two ROC curves. In **(D)**, the DeLong’s tests show that the *p*-value between the ROC curve of group 3 and the ROC curve of group 1 is less than 0.066, the *p*-value between the ROC curve of group 3 and the ROC curve of group 2 is less than 0.629, and the p-value between the ROC curve of group 2 and the ROC curve of group 1 is less than 0.030. In subfigure **(E)**, the DeLong’s tests show that the p-value between the ROC curve of group 3 and the ROC curve of group 1 is less than 0.003, the p-value between the ROC curve of group 3 and the ROC curve of group 2 is less than 0.037, and the *p*-value between the ROC curve of group 2 and the ROC curve of group 1 is less than 0.013. In **(F)**, the DeLong’s tests show that the *p*-value between the ROC curve of group 3 and the ROC curve of group 1 is less than 0.001, the *p*-value between the ROC curve of group 3 and the ROC curve of group *2* is less than 0.059, and the *p*-value between the ROC curve of group 2 and the ROC curve of group 1 is less than 0.003.

Next, we trained prediction models based on the radiomic signature and the clinical variables to approximately calculate the value of each unknown variable in Equations (1) and (2). We then validated the performance of these models on the independent validation set. The training results and the validation results are shown in **Table 3**. Of note, compared with A (arterial) phase and V (venous) phase, P (plain) phase obtained the best prediction performance for each group in the validation set. What is more, for each phase, we calculated the receiver operating characteristics curves (ROCs) and compared the ROCs of validation using the DeLong’s test method. **Figure 2** illustrates the ROC results. It demonstrates that the models based on the radiomic signature combined with clinical data (models based on groups 2 and 3) obtain better prediction results than the models based on the radiomic signature alone (models based on group 1). Additionally, although the indicator values based on group 3 show the highest performance, the model based on the radiomic signature combined with 4 clinical data in group 3 showed no significantly better prediction results in plain phase, compared with that combined with 2 clinical data in group 2 (*p* < 0.629). As can be seen from **Table 3**, the experiments based on plain phase obtained the best prediction performance than other phases. Thus, we also calculated the indicator values of accuracy, sensitivity, and specificity in the experiments of plain phase. **Table 4** and **Supplementary Figure S2** show the prediction results (on the validation set) as the threshold varied.

**Table 3:** Results of training and validation: plain (P), arterial (A), and venous (V); unless otherwise specified, the contents of parentheses are 95% confidence intervals.

Features	Training set (*n* = 83)	Validation set (*n* = 56)
**Group 1**		
P	0.911 (0.908–0.914)	**0.837 (0.827–0.847)**
A	0.913 (0.909–0.917)	0.710 (0.695–0.725)
V	0.874 (0.869–0.879)	0.625 (0.609–0.641)
**Group 2**		
P	0.919 (0.916–0.922)	**0.875 (0.867–0.883)**
A	0.895 (0.892–0.898)	0.783 (0.770–0.796)
V	0.900 (0.894–0.906)	0.742 (0.729–0.755)
**Group 3**		
P	0.895 (0.891–0.899)	**0.879 (0.869–0.889)**
A	0.892 (0.889–0.895)	0.828 (0.817–0.839)
V	0.902 (0.898–0.906)	0.797 (0.784–0.810)

group 1: radiomic signature; group 2: radiomic signature combining T stage and Dilated MPD/BD; group 3: radiomic signature combining T stage, Dilated MPD/BD, clinical TNM stage, and tumor margin. In the training, we used the fivefold crossvalidation technique to calculate the average AUC, then randomly performed 50 fivefold crossvalidations to calculate the average AUC and the 95% confidence intervals. In the independent validation, the bootstrap method based on sampling with replacement was used to calculate the average AUC and the 95% confidence intervals (based on 100 bootstraps). The sampling with replacement randomly sampled one sample at a time and drawn 56 times. The clinical TNM and T stages of the tumor were determined preoperatively according to the American Joint Committee on Cancer TNM Staging System Manual, 7th edition. Dilated MPD/BD, dilated main pancreatic duct (MPD) or bile duct (BD). The bold values in this table showed the best performance in each group.

**Table 4:** Validation results based on plain phase as the threshold varied: accuracy (ACC, %), sensitivity (SEN, %), and specificity (SPE, %).

Features	Threshold						
	0.5	0.55	0.6	0.65	0.7	0.75	0.8
Group 1							
ACC	75.0	75.0	76.8	78.6	78.6	**80.4**	76.8
SEN	83.8	83.8	83.8	81.1	78.4	**78.4**	73.0
SPE	57.9	57.9	63.2	73.7	78.9	**84.2**	84.2
Group 2							
ACC	75.0	78.6	82.1	**83.9**	80.4	80.4	82.1
SEN	83.8	83.8	83.8	**83.8**	78.4	78.4	75.7
SPE	57.9	68.4	78.9	**84.2**	84.2	84.2	94.7
Group 3							
ACC	78.6	76.8	80.4	**85.7**	85.7	83.9	80.4
SEN	86.5	83.8	83.8	**83.8**	83.8	81.1	73.0
SPE	63.2	63.2	73.7	**89.5**	89.5	89.5	94.7

group 1: radiomic signature; group 2: radiomic signature combining T stage and Dilated MPD/BD; group 3: radiomic signature combining T stage, Dilated MPD/BD, clinical TNM stage, and tumor margin. The clinical TNM and T stages of the tumor were determined preoperatively according to the American Joint Committee on Cancer TNM Staging System Manual, 7th edition. Dilated MPD/BD, dilated main pancreatic duct (MPD) or bile duct (BD). The bold values in this table showed the best performance in each group.

**Figure 3:**
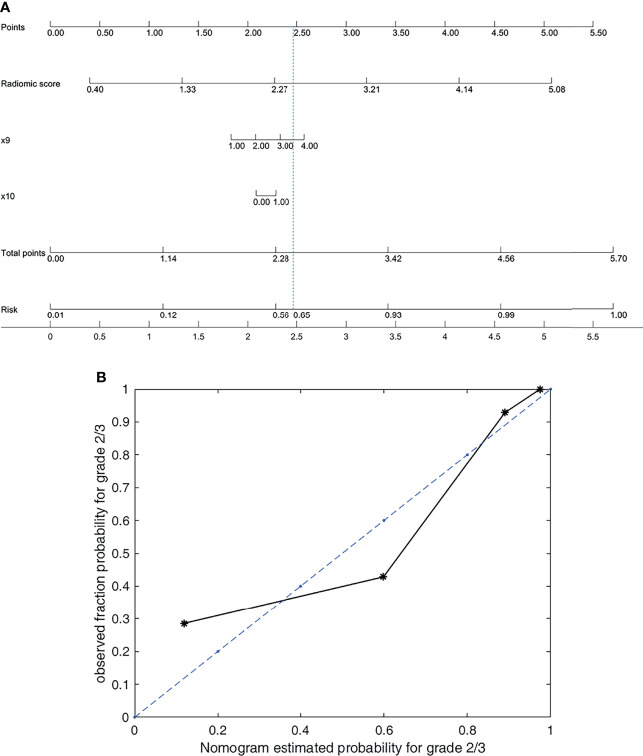
Nomogram and its calibration curve based on group 2 for predicting grade 2/3. **(A)** Nomogram for group 2. **(B)** Calibration curve, where the diagonal dotted line is a perfect estimation by an ideal model. The predicted (estimated) probabilities of the validation set were sorted and divided into four groups based on quartiles to calculate the observed true probabilities. We calculated the goodness of fit to evaluate how well the solid line fits the dotted line. The goodness of fit is 0.8683, which indicates that the two lines fit well.

Above all, the model based on the radiomic signature of plain phase combined with 2 clinical data (T stage and Dilated MPD/BD) in group 2 obtained the best prediction performance. Although the prediction results of the model based on the radiomic signature combined with 4 clinical data in group 3 seemed a little better, there was no significant differences between the groups 3 and 2 models (*p* < 0.629). Consider the balance between the convenience and predictive power of the model, we established a novel nomogram to preoperatively predict histologic grade in PNETs based on the radiomic signature of plain phase combined with 2 clinical data (T stage and Dilated MPD/BD) in group 2 (**Figure 3**).

**Figure 3** shows that the nomogram achieves a goodness of fit of 0.868 to the ideal model. Correspondingly, the score for the radiomic signature based on plain phase is


$$\begin{array}{l} y=\frac{x_{1}-217.2809}{6\times 167.0185}\times 1.943+\frac{x_{2}-0.9677}{6\times 0.0548}\times 3.1606+\frac{x_{3}-0.0956}{6\times 0.0264}\times \left(-2.4079\right)+\frac{x_{4}-546.9485}{6\times 143.0338}\times 2.7323+\\ \frac{x_{5}-1.0120}{6\times 0.1098}\times 1.0809+\frac{x_{6}-6.4072\times 10^{4}}{6\times 3.2953\times 10^{4}}\times \left(-37455\right)+\frac{x_{7}-117.4458}{6\times 17.8108}\times \left(-1.9601\right)+\frac{x_{8}-38.2048}{6\times 17.6032}\times 2.7947-0.6808 \end{array}$$

The score for the radiomic signature and clinical data in group 2 based on plain-phase is


$$\begin{array}{l} y=\frac{x_{1}-217.2809}{11.5\times 167.0185}\times 3.1548+\frac{x_{2}-0.9677}{11.5\times 0.0548}\times 3.4514+\frac{x_{3}-0.0956}{11.5\times 0.0264}\times \left(-3.5448\right)+\frac{x_{4}-546.9485}{11.5\times 143.0338}\times 3.6584+\\ \frac{x_{5}-1.0120}{11.5\times 0.1098}\times 1.55+\frac{x_{6}-6.4072\times 10^{4}}{11.5\times 3.2953\times 10^{4}}\times \left(-5.6929\right)+\frac{x_{7}-117.4458}{11.5\times 17.8108}\times \left(-2.5886\right)+\frac{x_{8}-38.2048}{11.5\times 17.6032}\times 4.335+\\ \frac{x_{10}-2.3133}{11.5\times 0.9097}\times \left(-2.6004\right)+\frac{x_{11}-0.2590}{11.5\times 0.4373}\times \left(-1.4059\right)-0.5775 \end{array}$$

The score for the radiomic signature and clinical data in group 3 based on plain phase is


$$\begin{array}{l} y=\frac{x_{1}217.2809}{7\times 167.0185}\times 1.463+\frac{x_{2}-0.9677}{7\times 0.0548}\times 1.2912+\frac{x_{3}-0.0956}{7\times 0.0264}\times \left(-1.5801\right)+\frac{x_{4}-546.9485}{7\times 143.0338}\times 1.1559+\\ \frac{x_{5}-1.0120}{7\times 0.1098}\times 1.1345+\frac{x_{6}-6.4072\times 10^{4}}{7\times 3.2953\times 10^{4}}\times \left(-2.3502\right)+\frac{x_{7}-117.4458}{7\times 17.8108}\times \left(-0.7985\right)+\frac{x_{8}-38.2048}{7\times 17.6032}\times 2.084+\\ \frac{x_{9}-2.4535}{7\times 0.9269}\times \left(-1.3666\right)+\frac{x_{10}-2.3133}{7\times 0.9097}\times \left(-1.2045\right)+\frac{x_{11}-0.2590}{7\times 0.4373}\times \left(-0.5704\right)+\frac{x_{12}-0.3554}{7\times 0.4784}\times \left(-1.1731\right)-0.595 \end{array}$$

## Discussion

PNETs are relatively rare neoplasms, the incidence of which is about 4–5 individuals per 100,000 annually (27). Nevertheless, PNETs have been increasingly detected and diagnosed in recent decades and currently represent the second most common pancreatic tumor followed by pancreatic adenocarcinoma (28). Most of PNETs carry MEN1, ATRX, or DAXX gene mutations, while approximately 15% activate mammalian target of rapamycin (mTOR) signaling (29, 30). PNETs are heterogenous neoplasms, of which the prognosis varies widely. The current most important prognostic stratification factor is WHO tumor grade classification, which might optimize tailored therapeutic strategies. So far, tumor grade is obtained by postoperative pathology. The preoperative fine-needle aspiration (FNA) is invasive, and the accuracy in differentiating tumor grade remains challenging. In the present study, we establish a combined nomogram that integrates a radiomic signature based on plain CT images with clinical features for noninvasive and preoperative prediction of pathologic grades of PNETs with high accuracy.

Firstly, we build a model based on an eight-feature radiomic signature to stratify PNET patients into G1 and G2/3 groups with an AUC of 0.911 (95% CI, 0.908–0.914) and 0.837 (95% CI, 0.827–0.847) in the training and validation cohorts, respectively. Moreover, we identified some objective clinical features (including T stage and dilated main pancreatic duct/bile duct status) related to tumor grade. Interestingly, the predictive performance was further improved by combining the radiomic signature with the clinical features mentioned above as a combined nomogram, achieving an AUC of 0.919 (95% CI, 0.916–0.922) and 0.875 (95% CI, 0.867–0.883) in the training and validation cohorts, respectively.

Recent developments in radiomics attract much interests in tumor detection, subtype classification, therapeutic response assessment, prediction of clinical outcome and tumor monitoring, etc. Most of them were attempt to stratify the biological behavior and optimize tailored therapeutic strategies for these heterogenous tumors such as PNETs. Traditional radiographic assessment [including CT (15) and MRI (17)] which commonly relies on visual evaluation, was previously reported to predict the biological aggressiveness of PNETs. Moreover, (18)F-FDG-PET/CT and (68)Ga-DOTANOC-PET/CT were reported to be useful in predicting tumor grade (14). However, the results vary a lot and the accuracy remains challenging, as the prediction was mainly established based on visual observation rather than quantitative information.

Radiomics and artificial intelligence (AI) automatically extract high-throughput quantitative image data. Just as limited studies reported previously, it could be more useful for differentiating pathologic grading in patients with PNETs than routine CT image features alone (31, 32). Whereas, combining feature engineering and machine learning is a widely used scheme in radiomics-aided diagnosis (32). Deep learning features are highly versatile, their ability to solve specific problems is relatively weak (33). In contrast, building an interpretable AI model based on feature engineering is relatively easy. The output of the model is expected to be understood by physicians in clinical applications. Nomograms based on linear models intuitively illustrate what drives the recognition in machine learning. We build a nomogram based on the group 2 model in plain phase, as shown in **Figure 3**. Wan’s research (34) investigated the performance of the combination of conventional handcrafted and learning-based features in disease recognition. For a specific research question, they emphasised that developing specific feature selection and model optimization approaches was necessary to achieve high accuracy and robustness. Consistent with this, the present paper proposed our optimized approaches according to the PNET grading issue (as illustrated in **Figure 1**).

As depicted in **Table 1**, our data showed that tumor size in grade 2/3 group was significantly larger than that in grade 1 group (*p* = 0.028). Dilated MPD/BD and poorly defined tumor margin were more frequently detected in patients with grade 2/3 PNETs than those with grade 1 (*p* = 0.02 and *p* = 0.013, respectively). Consistent results occurred in both training and validation sets in the present study. Moreover, research by Kim and colleague (15) identified the three indentical tumor CT features above as predictors of higher tumor grade of PNETs. Of note, the assessment of these features was relatively objective and the data can be automatically acquired in bulk. On the contrary, the data of tumor T stage, TNM stage, and diagnosis of insulinoma were obtained partly by doctors’ experience, although these features were suggested to be significantly different between grade 2/3 PNETs and grade 1 group in our study (**Table 1**). Therefore, to improve the predictive performance, we establish a combined nomogram model that integrates radiomic signature with the former three relatively objective clinical features (including tumor size, tumor margin, and dilated main pancreatic duct/bile duct) (**Table 2**). To our knowledge, our present comprehensive nomogram is the first study that integrates radiomic signature based on plain CT images with objective clinical features for noninvasive and preoperative prediction of pathologic grades for each PNET patients with high accuracy (both in training and independent validation set). Wenjie Liang and colleagues (35) reported a nomogram combining radiomic signature based on contrast-enhanced CT and clinical stage. Plain CT has lower cost and more convenience than contrast-enhanced CT. Also, accurate preoperative TNM staging of the tumor is difficult, as the preoperative assessment of “N” and “M” status remains challenging. Interestingly, Zhang’s research (36) depicted impressive results based on enhanced CT radiomic features with 3D modeling.

As is known to us, a quite different therapeutic strategy could be applied for PNETs of grades 1 and 2/3. For clinical practice, the present combined nomogram may facilitate personalized treatment decisions for each patient with this heterogeneous tumor. It is noninvasive and could identify PNETs of grades 1 and 2/3 with high accuracy preoperatively. According to ENET guidelines in terms of PNET, NF-PNETs of less than 2 cm with grade 1 were optimized candidates for a “wait and see” policy. Moreover, parenchyma-sparing procedure such as enucleation could be an alternative for PNET with grade 1, while radical resection with formal lymphadenectomy was recommended for PNET with grade 2/3. In addition, the therapeutic strategies for the advanced PNETs of graded 1 and 2/3 varied dramatically (palliative surgery, somatostatin analog, targeted therapy, or chemotherapy). Our present combined model may facilitate tailored surgical decisions. Additionally, given the spatial and temporal heterogeneity of the specific tumor, the noninvasive model can be used repeatedly for tumor monitoring (especially for the patient initially recommended to wait and see) and to dynamically optimize therapeutic regimen for patients with advanced PNETs.

A major limitation of the present study was the relatively insufficient sample size. In addition, given that the G3 group was small (approximately 10% of PNETs in our series and as previously reported), our present nomogram model was established to stratify PNET patients into G1 and G2/3 groups. To better optimize personalized therapeutic strategies, a nomogram to separate G2 and G3 groups is further needed to be established based on lager samples. We are trying to collect more cases from multicenters and explore more appropriate methods to conduct further studies. Thirdly, we used single-layer CT image in this study, while 3D modeling may more comprehensively reflect the overall characteristics of the tumor, it is worth exploring whether it can obtain a more powerful predictive capability. On the other hand, manual tumor segmentation for 3D modeling was time consuming, and it was not applied for small tumors without thin-slice CT scans.

## Conclusions

The developed combined nomogram that integrates radiomic signature based on plain CT images with clinical features (including T stage and dilated main pancreatic duct/bile duct status) can effectively predict the pathologic grades of PNETs preoperatively with powerful predictive capability. The noninvasive predictive model could assist clinicians to optimize tailored therapeutic strategies and facilitate surgical decision-making for each patient with PNETs in practice. It intuitively illustrates what drives the recognition in the prediction, which is potentially valuable in actual clinical applications and precision medicine in the future.

## Data Availability Statement

The original contributions presented in the study are included in the article/**Supplementary Material**. Further inquiries can be directed to the corresponding authors.

## Ethics Statement

Ethical review and approval was not required for the study on human participants in accordance with the local legislation and institutional requirements.

## Author Contributions

XW, J-JQ, DC, X-BL, and N-WK: study concept and design, data analysis and interpretation, and drafting of the manuscript. XW, J-JQ, C-LT, Q-QT, S-JR, and Y-HC: substantial contribution to data acquisition and interpretation and critical revision of the manuscript. XW and J-JQ: substantial contribution to data acquisition and data analysis. All authors: final approval of the manuscript and agreement with all the aspects of the work. All authors contributed to the article and approved the submitted version.

## Funding

This work was supported in part by research grants from the National Natural Science Foundation of China (82002579), the China Postdoctoral Science Foundation Funded Project (2019M663519), the Science and Technology Support Project of Sichuan Province (2020YFS0262), and the Post-Doctor Research Project, West China Hospital, Sichuan University (2019HXBH044).

## Conflict of Interest

The authors declare that the research was conducted in the absence of any commercial or financial relationships that could be construed as a potential conflict of interest.

## Publisher’s Note

All claims expressed in this article are solely those of the authors and do not necessarily represent those of their affiliated organizations, or those of the publisher, the editors and the reviewers. Any product that may be evaluated in this article, or claim that may be made by its manufacturer, is not guaranteed or endorsed by the publisher.
