# Supplementary material for: Development and Validation of a Novel Radiomics-Based Nomogram With Machine Learning to Preoperatively Predict Histologic Grade in Pancreatic Neuroendocrine Tumors
Source: Front Oncol. 2022 Mar 31;12:843376. doi: 10.3389/fonc.2022.843376 (PMC9008322; doi:10.3389/fonc.2022.843376)
Supplement: Supplementary file 1 [file DataSheet_1.docx]

# Supplemental Materials

## ROC curves of three phases in validation.


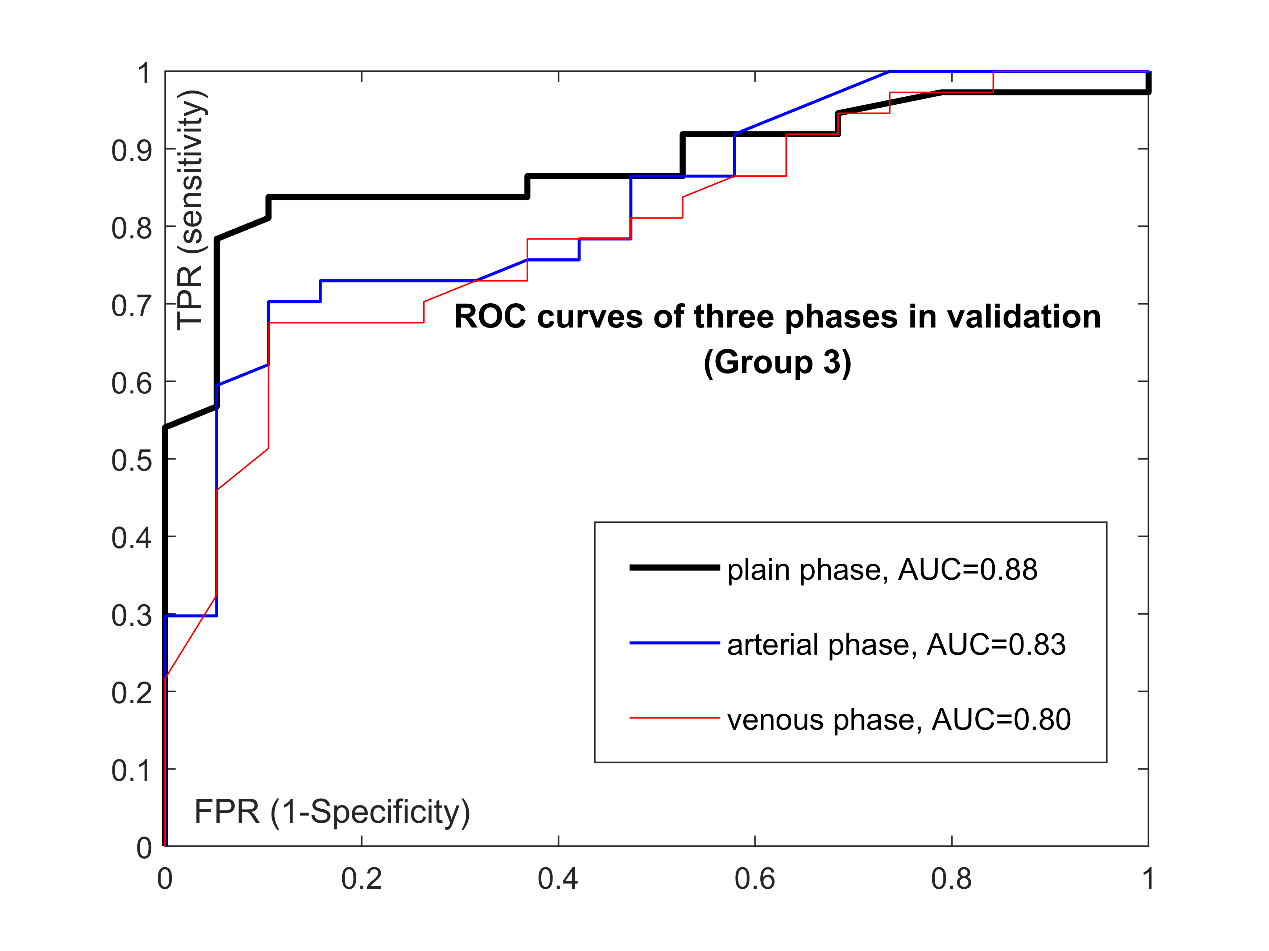
 p<0.287; p<0.144

Figure S1 ROC curves of three phases in validation. In each phase, the radiomic signature with clinic data in group 3 obtained the best performance. The DeLong’s tests shows that the p-value between the ROC curve of plain phase and the ROC curve of arterial phase is less than 0.287, the p-value between the ROC curve of plain phase and the ROC curve of venous phase is less than 0.144, and the p-value between the ROC curve of arterial phase and the ROC curve of venous is less than 0.485.

As for Group 3, there was no statistical difference between the ROCs in the arterial phase and the venous phase, and they were both worse than the ROC in the plain phase. The p-values for the plain phase vs. the arterial phase and the plain phase vs. the venous phase are 0.287 and 0.144, respectively. It indicates that the ROC of the plain phase is statistically better/different than that of the arterial phase and the venous phase, but it is not extremely significant. Considering indicators of accuracy, sensitivity, and specificity, the model performance of the plain phase is apparently better than that of the arterial phase and the venous phase. As for Group 1, the model performance of the plain phase is obviously better than that of the arterial phase and the venous phase.

## Texture Analysis Methods

Supplementary Table S1 shows the feature names extracted in this work.

Supplementary Table S1 Feature names of the texture analysis methods

| **No.** | **Method** | **Feature name** | **Number of features** |
| --- | --- | --- | --- |
| 1 | histogram | mean; variance; smoothness; skewness; uniformity; entropy; kurtosis; percentage of 0.01, 0.10, 0.50, 0.90, and 0.99; [1-2] | 12 |
| 2 | GLCM | energy (angular second moment); contrast; correlation; sum of squares (variance); homogeneity (inverse difference moment); sum average; sum variance; sum entropy; entropy; difference variance; difference entropy; information measure of correlation 1; information measure of correlation 2; maximal correlation coefficient; autocorrelation; dissimilarity; cluster shade; cluster prominence; maximum probability; inverse difference; inverse difference moment normalized; inverse difference normalized; Renyi entropy; Tsallis entropy; [3-7] | 24 (d=1) |
| 3 | GLRLM | short-run emphasis; long-run emphasis; gray-level nonuniformity; run-length nonuniformity; run percentage; low gray-level run emphasis; high gray-level run emphasis; short-run low gray-level emphasis; short-run high gray-level emphasis; long-run low gray-level emphasis; long-run high gray-level emphasis; [8] | 11 |
| 4 | LoG | mean; standard deviation; mean atte1nuation of pixels > 0; entropy; skewness; kurtosis; [9] | 36 (6 subbands) |
| 5 | wavelet transform | refer to the methods of histogram, GLCM, and GLRLM; [10] | 376 (8 components) |
| 6 | contourlet transform | refer to the methods of histogram, GLCM, and GLRLM; [11] | 611 (13 components) |
| 7 | ACM | refer to GLCM; [12] | 48 (d = 1, two gradients) |
| 8 | absolute gradient | mean; variance; skewness; kurtosis; non-zeros; [2] | 5 |
| 9 | autoregression | θ_1_, θ_2_, θ_3_, θ_4_, σ; [2] | 5 |
| 10 | GLDM | contrast, angular second moment, entropy, mean, inverse different moment; [2] | 5 (d = 1) |

Texture analysis methods were performed based on CT values. We calculated the histograms of ROIs in the training set and found that most of the pixels fell in [-34, 96]. Thus, we translated the ROIs into [1, 131].

Four directions (0, 45, 90, and 135) were averaged in the texture analysis methods involving GLCM, GLRLM, ACM, and GLDM. The distance was set to 1 the texture analysis methods involving GLCM, GLRLM, ACM, and GLDM.

The LoG method set sigma to 0,0.5, 1, 1.5, 2, and 2.5, so it derived 6 subbands. The wavelet transform method performed 2-level decomposition.

We tried 52 wavelets and calculated their entropies based on the training set. Finally, we selected the wavelet of db10 based on the maximum entropy. The wavelet transform derived 8 components from an ROI.

The contourlet transform chose the widely used 9-7 filter as the pyramid filter and the pvka6 filter as the directional filter. The contourlet transform also performed 2-level decomposition. The first decomposition derived 8 components and the second decomposition derived 4 components and an approximate component.

The ACM method decomposed an ROI into a direction gradient matrix and a magnitude gradient matrix.

## Feature Selection

We performed the Mann–Whitney U test (p-value <= 0.25) to preselect features. Then, we tried multiple algorithms, including the minimum redundancy maximum relevance (MRMR) algorithm [13], the infinite latent feature selection (ILFS) algorithm [14], the Chi-2 test algorithm, the Laplacian scoring algorithm, and the stepwise logistical regression algorithm, etc., to select the final features. Considering the number of training samples and the need to use an interpretable classification model to make predictions, the final number of features was limited to 8. Based on the final features of each feature selection algorithm, we built an SVM-linear model and a logistic regression model on the training set, and then performed a 5-fold cross-validation. AUCs were calculated and compared to determine which feature selection algorithm was optimal. Finally, we combined the LASSO (Least absolute shrinkage and selection operator) algorithm and the stepwise logistical regression to select features. In the LASSO algorithm, MSE (minimum cross-validated mean squared error) was calculated as an indicator to select candidate λ values. As for a λ value, we screened out those features corresponding to non-zero coefficients, and then built a stepwise logistic regression model to further reduce the number of features. R-squared was calculated in the stepwise logistic regression. Those features corresponding to the maximum R-squared were finally selected.

## Classification Tasks

Considering the number of training samples and the need to use an interpretable classification model to make predictions, we tried two widely used linear machine learning models: SVM-linear and LR (logistical regression). In training, their classification results were very close, where SVM-linear was slightly better. Thus, we selected SVM-linear models to perform classification results. Supplementary Table S3 shows the results of 5-fold cross-validation in the training.

**Supplementary Table S2** Training results of SVM-linear models and LR models. P: plain; A: arterial; V: venous.

| **Feature group** | **Phase** | **AUC of SVM-linear** | **AUC of LR** |
| --- | --- | --- | --- |
| Group 1 | P | 0.911 (0.908-0.914) | 0.893 (0.886-0.900) |
|  | A | 0.913 (0.909-0.917) | 0.908 (0.904-0.912) |
|  | V | 0.874 (0.869-0.879) | 0.884 (0.878-0.890) |
| Group 2 | P | 0.919 (0.916-0.922) | 0.881 (0.873-0.889) |
|  | A | 0.895 (0.892-0.898) | 0.890 (0.885-0.895) |
|  | **V** | 0.900 (0.894-0.906) | 0.895 (0.887-0.903) |
| Group 3 | P | 0.895 (0.891-0.899) | 0.848 (0.839-0.857) |
|  | A | 0.892 (0.889-0.895) | 0.895 (0.890-0.900) |
|  | **V** | 0.902 (0.898-0.906) | 0.893 (0.883-0.903) |

Footnote: Radiomic signature (group 1); Radiomic signature combining T stage and Dialated main pancreatic duct (MPD) / bile duct (BD) (group 2); Radiomic signature combining T stage, Dialated MPD/BD, Clinical TNM stage, and Tumor margin (group 3). In the training, we used the 5-fold cross-validation technique to calculate the average AUC, then randomly performed 50 5-fold cross-validations to calculate the average AUC, its 95% confidence interval.


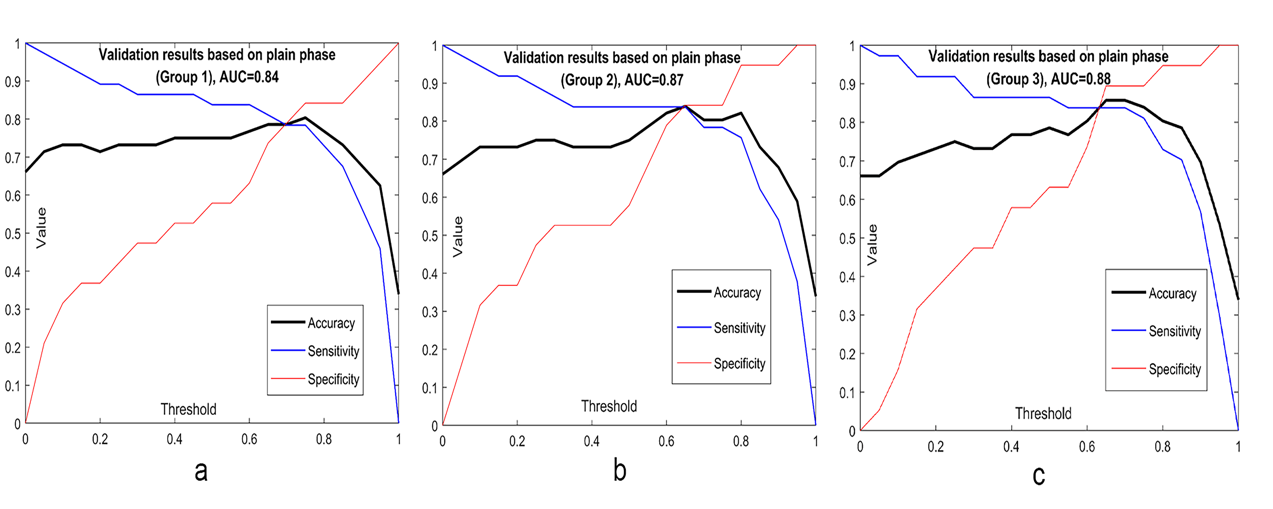


**Figure S2**

Plain-phase based validation results of accuracy, sensitivity, and specificity as the threshold varied: (a) validation results based on Group 1; (b) validation results based on Group 2; (c) validation results based on Group 3

1. Gonzalez R C, Woods R E, Eddins S L. Digital Image Processing Using Matlab, 2nd edn. Gatesmark Publishing, Knoxville, 2009, pp 644-656.
2. Szczypiński P M, Strzelecki M, Materka A, et al. MaZda—a software package for image texture analysis[J]. Computer methods and programs in biomedicine, 2009, 94(1): 66-76.
3. Haralick R M, Shanmugam K, Dinstein I H. Textural features for image classification[J]. IEEE Transactions on systems, man, and cybernetics, 1973 (6): 610-621.
4. Soh L K, Tsatsoulis C. Texture analysis of SAR sea ice imagery using gray level co-occurrence matrices[J]. IEEE Transactions on geoscience and remote sensing, 1999, 37(2): 780-795.
5. Clausi D A. An analysis of co-occurrence texture statistics as a function of grey level quantization[J]. Canadian Journal of remote sensing, 2002, 28(1): 45-62.
6. Banik S, Rangayyan R M, Desautels J E L. Measures of angular spread and entropy for the detection of architectural distortion in prior mammograms[J]. International journal of computer assisted radiology and surgery, 2013, 8(1): 121-134.
7. Yang X, Tridandapani S, Beitler J J, et al. Ultrasound GLCM texture analysis of radiation‐induced parotid‐gland injury in head‐and‐neck cancer radiotherapy: An in vivo study of late toxicity[J]. Medical physics, 2012, 39(9): 5732-5739.
8. Tang X. Texture information in run-length matrices[J]. IEEE transactions on image processing, 1998, 7(11): 1602-1609.
9. Gunn S R. On the discrete representation of the Laplacian of Gaussian[J]. Pattern Recognition, 1999, 32(8): 1463-1472.
10. Mallat S G. A theory for multiresolution signal decomposition: the wavelet representation[J]. IEEE transactions on pattern analysis and machine intelligence, 1989, 11(7): 674-693.
11. Do M N, Vetterli M. The contourlet transform: an efficient directional multiresolution image representation[J]. IEEE Transactions on image processing, 2005, 14(12): 2091-2106.
12. Chakraborty J, Midya A, Mukhopadhyay S, et al. Automatic characterization of masses in mammograms[C]//2013 6th International Conference on Biomedical Engineering and Informatics. IEEE, 2013: 111-115.
13. Ding C, Peng H. Minimum redundancy feature selection from microarray gene expression data[J]. Journal of bioinformatics and computational biology, 2005, 3(02): 185-205.
14. Roffo G, Melzi S, Castellani U, et al. Infinite latent feature selection: A probabilistic latent graph-based ranking approach[C]//Proceedings of the IEEE International Conference on Computer Vision. 2017: 1398-1406.
